# Assessment of Patient Ambulation Profiles to Predict Hospital Readmission, Discharge Location, and Length of Stay in a Cardiac Surgery Progressive Care Unit

**DOI:** 10.1001/jamanetworkopen.2020.1074

**Published:** 2020-03-17

**Authors:** In cheol Jeong, Ryan Healy, Benjamin Bao, William Xie, Tim Madeira, Marc Sussman, Glenn Whitman, Jennifer Schrack, Nicole Zahradka, Erik Hoyer, Charles Brown, Peter C. Searson

**Affiliations:** 1 inHealth, Johns Hopkins Individualized Health Initiative, Johns Hopkins University School of Medicine, Baltimore, Maryland; 2 Department of Critical Care and Anesthesiology, Johns Hopkins University School of Medicine, Baltimore, Maryland; 3 Department of Biomedical Engineering, Johns Hopkins University, Baltimore, Maryland; 4 Department of Computer Science, Johns Hopkins University School of Medicine, Baltimore, Maryland; 5 Department of Surgery, Johns Hopkins University School of Medicine, Baltimore, Maryland; 6 Department of Epidemiology, Johns Hopkins University Bloomberg School of Public Health, Baltimore, Maryland; 7 Department of Physical Medicine and Rehabilitation, Johns Hopkins University School of Medicine, Baltimore, Maryland; 8 Department of Materials Science and Engineering, Johns Hopkins University, Baltimore, Maryland

## Abstract

**Importance:**

Promoting patient mobility during hospitalization is associated with improved outcomes and reduced risk of hospitalization-associated functional decline. Therefore, accurate measurement of mobility with high–information content data may be key to improved risk prediction models, identification of at-risk patients, and the development of interventions to improve outcomes. Remote monitoring enables measurement of multiple ambulation metrics incorporating both distance and speed.

**Objective:**

To evaluate novel ambulation metrics in predicting 30-day readmission rates, discharge location, and length of stay using a real-time location system to continuously monitor the voluntary ambulations of postoperative cardiac surgery patients.

**Design, Setting, and Participants:**

This prognostic cohort study of the mobility of 100 patients after cardiac surgery in a progressive care unit at Johns Hopkins Hospital was performed using a real-time location system. Enrollment occurred between August 29, 2016, and April 4, 2018. Data analysis was performed from June 2018 to December 2019.

**Main Outcomes and Measures:**

Outcome measures included 30-day readmission, discharge location, and length of stay. Digital records of all voluntary ambulations were created where each ambulation consisted of multiple segments defined by distance and speed. Ambulation profiles consisted of 19 parameters derived from the digital ambulation records.

**Results:**

A total of 100 patients (81 men [81%]; mean [SD] age, 63.1 [11.6] years) were evaluated. Distance and speed were recorded for more than 14 000 segments in 840 voluntary ambulations, corresponding to a total of 127.8 km (79.4 miles) using a real-time location system. Patient ambulation profiles were predictive of 30-day readmission (sensitivity, 86.7%; specificity, 88.2%; C statistic, 0.925 [95% CI, 0.836-1.000]), discharge to acute rehabilitation (sensitivity, 84.6%; specificity, 86.4%; C statistic, 0.930 [95% CI, 0.855-1.000]), and length of stay (correlation coefficient, 0.927).

**Conclusions and Relevance:**

Remote monitoring provides a high–information content description of mobility, incorporating elements of step count (ambulation distance and related parameters), gait speed (ambulation speed and related parameters), frequency of ambulation, and changes in parameters on successive ambulations. Ambulation profiles incorporating multiple aspects of mobility enables accurate prediction of clinically relevant outcomes.

## Key Points

**Question:**

Are patient ambulation profiles predictive of hospital readmission, discharge location, and length of stay?

**Findings:**

In this prognostic cohort study of 100 adults in a cardiac surgery progressive care unit, patient ambulation profiles were predictive of 30-day readmission (C statistic, 0.925), discharge location (C statistic, 0.930), and length of stay (correlation coefficient, 0.927).

**Meaning:**

Patient ambulation profiles from a real-time location system enable prediction of clinically relevant outcomes.

## Teaser

This prognostic cohort study evaluates novel ambulation metrics in predicting 30-day readmission rates, discharge location, and length of stay using a real-time location system to continuously monitor the voluntary ambulations of postoperative cardiac surgery patients.

## Introduction

Promoting patient mobility is recognized as an important strategy to decrease the risk of hospitalization-associated functional decline and to improve postoperative recovery.^1,2,3,4^ However, accurate measurement of mobility over time in the hospital remains a major roadblock to developing accurate risk prediction models, identifying at-risk patients, and developing interventions to improve outcomes.^5^

Although there are many methods to assess mobility, each has advantages and limitations. Mobility questionnaires provide a snapshot of a patient’s functional status but are subjective and difficult to measure continuously. Timed-walk tests provide information on distance and speed^6,7^ but are labor intensive and, hence, are impractical to implement multiple times during hospitalization. Nonetheless, studies^8,9^ in different patient populations using these methods have highlighted the potential in predicting outcomes such as length of stay. Wearable accelerometer-based sensors enable continuous monitoring of patient ambulation,^10^ although validation and standardization in individuals who walk slowly and/or with abnormal gait patterns remains a challenge.^11,12,13,14,15^ Despite these limitations, studies^10,16,17^ of hospitalized patients with wearable accelerometers have found statistically significant differences in patient outcomes (eg, length of stay, 30-day readmission, and patient disposition) based on daily step counts. However, estimations of outcomes are modest at best, with areas under the curve of approximately 0.7. Although these studies have largely focused on step count, it is not known which ambulation metrics are most predictive of patient outcomes. Although daily step counts enable continuous monitoring, additional parameters, such as ambulation speed and the change in distance and speed during recovery, could refine assessment of patient mobility and improve prediction models. The objectives of this study were to use a real-time location system to continuously monitor voluntary out-of-room ambulations of postoperative cardiac surgery patients and to assess multiple ambulation metrics in predicting readmission rate, discharge location, and length of stay.

## Methods

### Study Design and Participants

This prognostic cohort study was approved by the Johns Hopkins institutional review board as a research project, and all patients provided written informed consent. This study follows Strengthening the Reporting of Observational Studies in Epidemiology (STROBE) reporting guideline for cohort studies and the Transparent Reporting of a Multivariable Prediction Model for Individual Prognosis or Diagnosis (TRIPOD) reporting guideline.

The study was performed in a 33-bed cardiac surgery progressive care unit (PCU) at Johns Hopkins Hospital with patients undergoing surgery between August 29, 2016, and April 4, 2018 (Table; eAppendix 1 in the Supplement). Eligibility was determined through review of the electronic health record.

**Table.** Patient and Surgical Characteristics

Characteristic	Cardiac Surgery Patients, No. (%) (N = 100)
Age, median (IQR), y	65 (58-72)
Male	81 (81)
Race	
White	82 (82)
African American	11 (11)
Other	7 (7)
Comorbidities	
Prior stroke	6 (6)
Hypertension	84 (84)
Congestive heart failure	25 (25)
Peripheral vascular disease	12 (12)
Chronic obstructive pulmonary disease	12 (12)
Tobacco use (current)	17 (17)
Diabetes	43 (43)
European System for Cardiac Operative Risk Evaluation log score, median (IQR)	2.49 (1.9-4.0)
Surgery	
Coronary artery bypass only	64 (64)
Valve only	17 (17)
Other	19 (19)
Cardiopulmonary bypass duration, median (IQR), min	99 (75-125)

Abbreviation: IQR, interquartile range.

### Ambulation Profiles

**Figure 1.**
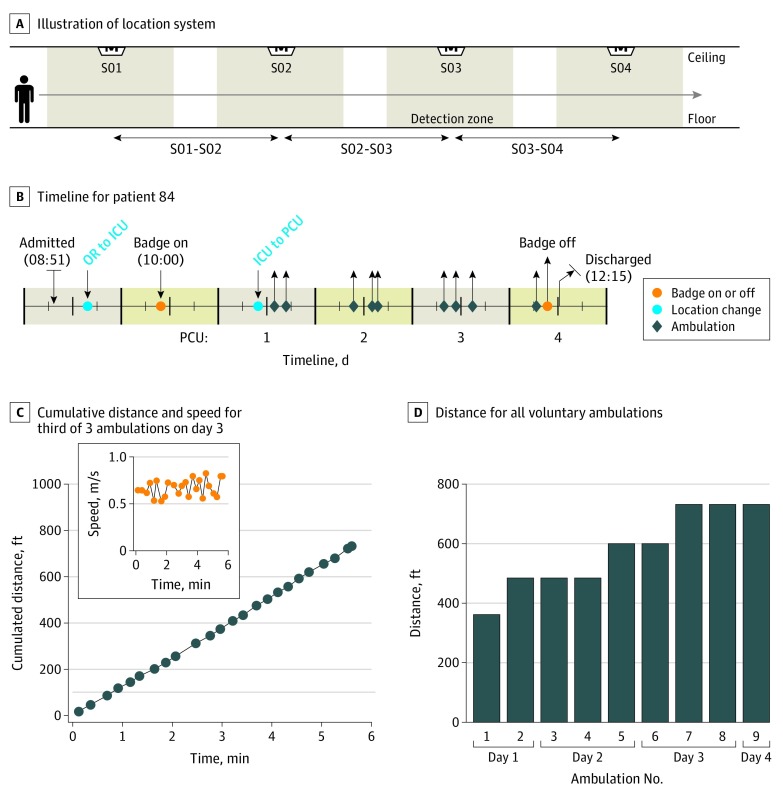
Remote Monitoring of Patient Ambulation A, Schematic illustration of the infrared real-time location system. An infrared badge, worn by the patient, emits an infrared signal that is detected by ceiling sensors in the corridors and patient rooms. Detection of a badge signal by a ceiling sensor results in transmission of the badge identification, the sensor location, and a time stamp to a server. B, Timeline for patient 84. This patient was in the progressive care unit (PCU) for 4 days and performed 9 voluntary ambulations before discharge. C, Cumulative distance for the third of 3 ambulations on day 3. The inset shows the speed for each segment. D, Distance for all voluntary ambulations. To convert feet to meters, multiply by 0.3. ICU indicates intensive care unit; and OR, operating room.

Patient out-of-room ambulations were measured using an infrared (IR) real-time location system (RTLS; Midmark) designed for location of staff and equipment. An IR badge worn by an individual emits an IR signal that is detected by ceiling sensors (Figure 1A). The sensors are located at approximately 6-m (20-ft) intervals in the corridors, and the diameter of the detection zone is approximately 3.5 m (11 ft).^18^ Detection of a badge signal by a ceiling sensor results in transmission of the badge identification number, sensor location, and a time stamp to a server. We have previously validated the system for ambulation monitoring in a timed-walk test.^18^

The IR badges were attached to the patient’s gown after leaving the operating room or on the first postoperative day. From the badge data for each patient, ambulation maps were assembled from the individual segments corresponding to detection by successive ceiling sensors. We defined an ambulation as an event where a badge was detected by at least 6 ceiling sensors in the corridor before returning to the patient’s room. Each ambulation contains a set of segments, defined by distance and speed, that start and end at the patient’s room. A patient’s ambulation record comprised the information from all ambulations, from the day of transfer to the PCU to the day of discharge. Digital ambulation profiles for each patient included 19 metrics (eAppendix 2 in the Supplement) associated with the total number of ambulations, ambulation frequency, and the distance, duration, and speed associated with each ambulation (see eTable 1 and eTable 2 in the Supplement for examples). To account for the fact that transfer and discharge days often reduced the opportunity for ambulation, parameters were calculated on the basis of the total number of days and the number of full days (total number of days excluding the transfer and discharge days) in the PCU.

### Outcomes

Discharge location (acute or subacute rehabilitation or home) and length of stay, defined as the total number of days in the PCU, were extracted from the electronic health record. No patients were institutionalized at baseline. Patients who were discharged to a hotel or a family member’s home were considered to be discharged home for analytical purposes. Information on 30-day readmission was extracted from the electronic health record, which included information on readmissions outside of Johns Hopkins. Readmission was defined as any overnight stay past midnight in a hospital within 30 days of discharge from the initial procedure. Because adjudication of observation status or readmission is not readily apparent in the electronic health record, patients were considered to be readmitted if they were in a hospital (including the emergency department) past midnight. This approach was chosen to identify clinically relevant cases of patients needing hospital-level medical care, without administrative or technical exclusions that can lead to underreporting. Administrative data on readmissions were obtained to supplement the research classification.

### Statistical Analysis

Here we report data for all 100 patients who had their IR badge at discharge from the PCU and who recorded at least 1 voluntary ambulation (eAppendix 1 in the Supplement). To compare pairwise differences in outcomes between groups (yes or no readmission; yes or no discharge to acute or subacute rehabilitation or home) for each ambulation parameter and length of stay, we used 1-way analysis of variance to determine *F* scores and 2-sided *P* values. *P* < .05 was considered statistically significant. Predictions of outcomes (readmission, discharge disposition, and length of stay) were performed using linear regression with split sampling in SPSS statistical software version 26 (IBM).^19^ All models were created using the 19 ambulation parameters for 100 patients. No other patient-specific demographic or clinical variables were used. Because the number of adverse events was small, we first used a synthetic minority oversampling technique (imblearn.over_sampling.SMOTE algorithm in Python version 3.7.0, Python) to balance the data before analysis.^20^ The SMOTE algorithm is widely used and has been validated in various domains. After balancing, the data were split (70/30) into independent training and test sets, using the random number generator and compute variable functions. Predictions of 30-day readmission and discharge disposition (dependent variables) were performed using the binary logistic regression model with the 19 ambulation parameters and length of stay for all patients as covariates. Prediction of length of stay used the linear regression model, with 19 ambulation parameters as covariates. Receiver operating characteristic curves were created on the basis of the predicted outcomes (readmission and discharge location) from the test set and the corresponding outcomes from the balanced data set (receiving operating characteristic analysis module). Data analysis was performed from June 2018 to December 2019.

## Results

From August 29, 2016, to April 4, 2018, 238 patients consented to wear an IR badge to monitor voluntary out-of-room ambulations while in the PCU. Analysis was performed for 100 patients (19 women [19%] and 81 men [81%]) (Table; eAppendix 1 in the Supplement). Overall, the mean (SD) age of patients in the study was 63.1 (11.6) years, and the mean (SD) length of stay in the PCU was 5.9 (2.2) days. The 30-day readmission rate was 21% (21 of 100 patients), and 11 (11%) of the patients were discharged to acute or subacute rehabilitation.

Ambulation records were obtained for 100 patients during their stay in the PCU. Each voluntary out-of-room ambulation consists of a series of segments identified from detection of the patient’s badge by ceiling sensors (Figure 1A). The segments trace the route for each ambulation, from which the distance and speed can be calculated. Overall, we recorded more than 14 000 segments in 840 voluntary ambulations corresponding to a total of 127.8 km (79.4 miles). Figure 1B shows a representative timeline for a patient during hospitalization. This patient was in the PCU for 4 days and during this time completed 9 voluntary ambulations. The patient was discharged to home and was not readmitted within 30 days. For the example ambulation shown in Figure 1C, the ambulation distance increased linearly with time, indicating that the patient maintained a constant speed. In this ambulation, the patient walked approximately 229 m (750 ft) at a mean speed of approximately 0.7 m/s (Figure 1C). Ambulations can be summarized in terms of daily values, and for this patient, the total daily ambulation distance increased from approximately 244 m (800 ft) on the day of transfer to the PCU (day 1) to approximately 610 m (2000 ft) on day 3 (Figure 1D). In this study, very few patients exceeded a speed of 0.6 m/s in a single ambulation.

Ambulation profiles were created for each patient and included 19 metrics associated with each voluntary ambulation (see eAppendix 2 in the Supplement). Examples of ambulation record and profile for a patient who was not readmitted are provided in eFigure 1 and eTable 1 in the Supplement, and those for a patient who was readmitted within 30 days are shown in eFigure 2 and eTable 2 in the Supplement. Summaries of ambulation profile data for all patients are shown in eTable 3 and eTable 4 in the Supplement. The ambulation profiles provide high–information content data describing a patient’s ambulation status and trajectory.

**Figure 2.**
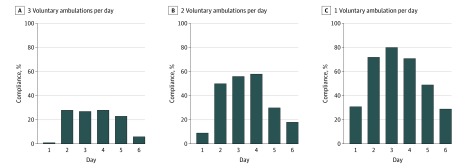
Compliance With Number of Completed Voluntary Ambulations per Day A-C, Graphs show data for 3 voluntary ambulations per day (A), 2 voluntary ambulations per day (B), and 1 voluntary ambulation per day (C). The mean (SD) length of stay was 5.9 (2.2) days. The total numbers of individuals were 100 at days 1, 2, and 3; 92 at day 4; 73 at day 5; and 47 at day 6. Data are shown for up to day 6 in the cardiac surgery progressive care unit.

While they are in the PCU, patients are encouraged to complete 3 voluntary ambulations per day. From the ambulation profiles, we found that the compliance rate with 3 ambulations per day was 28.0% on day 2 (the first full day after transfer to the PCU), 27.0% on day 3, and 28.0% on day 4 (Figure 2). The ability to remotely track simple metrics, such as compliance with voluntary ambulations, highlights a major advantage of remote monitoring systems.

**Figure 3.**
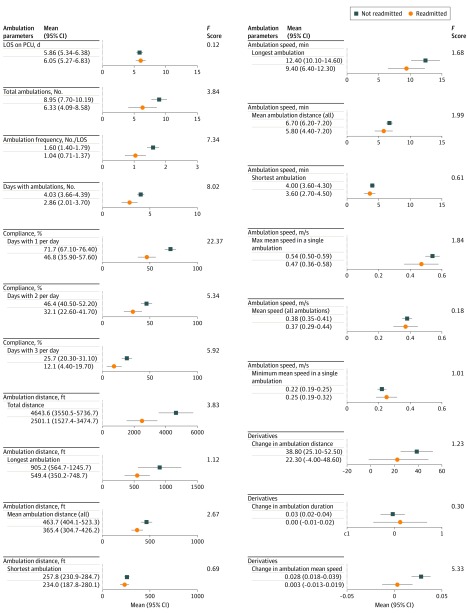
Comparison of Ambulation Parameters for 30-Day Readmission Comparisons are based on total days in the cardiac surgery progressive care unit (PCU) for 100 patients. To convert feet to meters, multiply by 0.3. LOS indicates length of stay.

To determine the association with clinically relevant outcomes, ambulation parameters were compared with 30-day readmission and discharge location (Figure 3; eFigure 3 and eTable 5 in the Supplement). Several ambulation parameters were statistically significant between the 2 groups (30-day readmission yes or no; discharge location yes or no). For 30-day readmissions, the 95% confidence limits for 4 ambulation parameters showed no overlap between the group not readmitted and the group readmitted: (1) mean number of ambulations per day (ambulation frequency) (1.60 [95% CI, 1.40-1.79] vs 1.04 [95% CI, 0.71-1.37]; *F* = 7.34; *P* = .008), (2) percentage of days with at least 1 ambulation (71.7% [95% CI, 67.1%-76.4%] vs 46.8% [95% CI, 35.9%-57.6%]; *F* = 22.37; *P* < .001), (3) percentage of days with at least 3 ambulations (25.7% [95% CI, 20.3%-31.1%] vs 12.1% [95% CI, 4.4%-19.7%]; *F* = 5.92; *P* = .03), and (4) total cumulative distance for all ambulations (4643.6 [95% CI, 3550.5-5736.7] vs 2501.1 [95% CI, 1527.4-3474.7] ft; *F* = 3.83; *P* = .05). Several other parameters were statistically significant, including total number of ambulations, number of days with ambulations, percentage of days with at least 2 ambulations, and change in mean ambulation speed (eTable 5 in the Supplement).

For discharge location, the 95% confidence limits for 1 parameter showed no overlap: the length of stay in the PCU (5.60 [5.20-5.99] vs 8.36 [6.36-10.36] days; *F* = 18.2; *P* < .001) (eFigure 3 in the Supplement). Other parameters that were statistically significant include percentage of days with at least 1 ambulation, percentage of days with at least 2 ambulations, and maximum speed in a single ambulation (eTable 5 in the Supplement).

To gain insight into a patient’s recovery trajectory, we calculated how mean distance, speed, and time changed between successive ambulations (eFigure 4 and eFigure 5 in the Supplement). The trajectory is defined in terms of the derivative of distance, speed, and time with respect to ambulation number. For individuals who were discharged home and were not readmitted, the increase in mean distance in successive ambulations was positive (eTable 3 and eTable 4 in the Supplement). However, the mean change in ambulation time was approximately 0, illustrating that there was little change in ambulation duration, but because their distance increased, there was a statistically significant increase in their ambulation speed compared with individuals who were readmitted (*P* = .02; *F* = 5.3). Therefore, the rate of change in ambulation speed was an important metric in recovery. For individuals who were readmitted or discharged to acute or subacute rehabilitation, the derivative in distance was generally small and positive, indicating that few patients showed a decrease in mobility during their stay in the unit.

**Figure 4.**
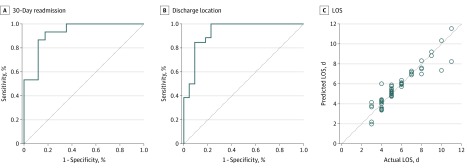
Receiver Operating Characteristic (ROC) Curves for 30-Day Readmissions and Discharge Location, and Predictions of Length of Stay (LOS) A, ROC curve for 30-day readmission (C statistic, 0.925; 95% CI, 0.836-1.000). B, ROC curve for discharge location (C statistic, 0.930; 95% CI, 0.855-1.000). The area under the ROC curve was calculated using a nonparametric (distribution-free) method. C, ROC curve for LOS. Models were generated using split sampling (70/30) following balancing based on data for the total number of days on the unit. Data shown are for the test set.

Having established that several ambulation parameters were statistically significant between 30-day readmission (yes or no) and discharge location (acute or subacute rehabilitation or home) groups, a binary linear regression model with split sampling was used to assess outcome predictions on the basis of the ambulation profiles (eTable 6 in the Supplement). Thirty-day readmissions were predicted with 86.7% sensitivity and 88.2% specificity, and the discharge location was predicted with 84.6% sensitivity and 86.4% specificity. The model predictions were similar according to full days in the PCU (eTable 6 in the Supplement). Receiver operating characteristic curves for the regression models (Figure 4) had area under the curve (C statistic) values of 0.925 (95% CI, 0.836-1.000) for 30-day readmissions and 0.930 (95% CI, 0.855-1.000) for discharge location. Next we assessed predictions of length of stay with ambulation profiles. The correlation coefficient for predicted of length of stay was 0.927 (see eTable 7 in the Supplement for details). In general, outcomes were improved in patients with at least 1 voluntary ambulation per day, a cumulative total ambulation distance of more than 1082 meters (3550 ft; ie, >7 laps around the PCU), a change in ambulation speed of more than 0.018 m/s per ambulation, and a length of stay of less than 6 days.

## Discussion

The findings of this study suggest that a real-time location system, already installed in the hospital for tracking the locations of staff and equipment, can be used to monitor voluntary out-of-room ambulations in a cardiac surgery PCU. From the ambulation data, we defined a patient’s digital ambulation profile, which included 19 ambulation parameters (eg, number, frequency, distance, and speed) and enabled predictions of outcomes.

Patient mobility is an important biomarker associated with postoperative recovery; however, accurate measurement remains challenging. A systematic review of risk prediction models for hospital readmissions, based solely on clinical parameters, concluded that they generally perform poorly,^5^ highlighting the need for measurement tools that have high information content.

Remote tracking and wearable accelerometers both provide measures of mobility but have some key differences: remote tracking can distinguish out-of-room ambulation events and can provide information on patient speed, although the resolution (approximately 6 m [20 ft] in this study) is larger than a single step. Furthermore, remote tracking is easy to implement and is scalable.

To promote mobility, patients in the PCU are encouraged to walk 3 times per day as part of the Activity & Mobility Promotion program.^9^ We found that the maximum compliance, on the first full day after transfer to the PCU, was 28.0%, and decreased on subsequent days, largely because the more ambulatory patients were generally discharged sooner (mean [SD] length of stay, 5.9 [2.2] days). Nonetheless, these results highlight the value of remote monitoring in assessing compliance to mobility guidelines. In general, outcomes were improved in patients with at least 1 voluntary ambulation per day, a cumulative total ambulation distance of more than 1082 meters (3550 ft; ie, >7 laps around the PCU), a change in ambulation speed of more than 0.018 m/s per ambulation, and a length of stay of less than 6 days.

Studies of hospitalized patients with wearable accelerometers have found statistically significant differences in patient outcomes with daily step counts. One study^16^ found that 55% of participants with fewer than 900 steps per day experienced hospitalization-acquired functional decline, compared with 18% of participants with 900 or more steps per day. Another study^17^ of postsurgical patients showed that the numbers of steps on the second, third, and fourth recovery days were statistically different between patients discharged to home compared with those discharged to a skilled nursing facility or home with health care. A third study^10^ showed that a threshold of 275 steps per day enabled prediction of 30-day readmission with a sensitivity of 42% and a specificity of 78%.

Although step count is a convenient metric, it does not provide information about speed and only provides an indirect measurement of distance. Several groups have shown that a short (4 or 6 m) gait speed test is correlated with survival in elderly adults,^21,22^ and thresholds for defining dismobility (very low gait speeds) of 0.6 to 0.8 m/s (1.34-1.70 mph) have been proposed for the general population. In the present study of a postsurgical population, very few patients exceeded 0.6 m/s in a single ambulation. However, all individuals who were not readmitted and discharged home showed a significant increase in speed in successive ambulations.

Prior studies^10,16,17,21,22^ highlight the fact that step count and gait speed are associated with clinical outcomes; however, our results show that more detailed measurements of mobility with higher information content enable a significant increase in predictive power. We incorporate elements of step count (ambulation distance and related parameters) and gait speed (ambulation speed and related parameters), as well as parameters associated with frequency, and changes in these parameters in successive ambulations. Together, these parameters enable improved predictions of clinically relevant outcomes. Comparison of our results with previous studies also suggest that the criteria for identification of at-risk patients is likely not generalizable and is different for different patient populations.

The real-time location system enables accurate reconstruction of the individual segments that constitute an ambulation. Our algorithms take into account anomalies, such as a missing sensor in a sequence that may occur if the badge is obstructed during that segment. Although there are sensors in patient rooms, the technology does not enable assessment of in-room activity. Here we arbitrarily define an ambulation as an event that begins and ends in a patient room, without leaving the unit. We ignored events where a patient left the unit, either on a voluntary ambulation or for medical reasons. A complication associated with leaving the unit for a test is that we cannot, at present, distinguish between ambulation and transport by wheelchair. Because data can be collected and analyzed in real-time, a patient’s ambulation history could be updated and displayed in the electronic medical record or on a patient’s smartphone in real time. The ability to track and monitor ambulation history could enable goal setting and identification of at-risk patients based on real-time prediction models.

### Limitations

This study has some limitations. It is a prognostic cohort study that was performed in a cardiac surgery PCU with a small sample size. Enrollment criteria were broad; hence, our results can be considered broadly generalizable. Data were analyzed for all patients who had their IR badge at discharge from the PCU and recorded at least 1 voluntary ambulation. Exclusion due to a lost or removed badge or failure to register at least 1 voluntary ambulation may have resulted in a selection bias. To improve patient compliance, alternative methods for wearing the IR badge should be explored. Exclusion of out-of-unit ambulations may have led to underestimating the total number of ambulations. Further studies will also be needed to assess the technology in other patient populations. Outcomes predictions were based solely on metrics from voluntary out-of-room ambulations; however, measurement of in-room activity and input from other patient-specific demographic or clinical variables could provide additional insight into patient functional status and outcomes.

## Conclusions

Real-time location system technology was used to monitor voluntary ambulations of patients in a cardiac surgery PCU. Each ambulation contains information on distance and speed, whereas a patient’s ambulation history provides insight into ambulation frequency and changes in ambulation metrics. Metrics associated with voluntary ambulations were predictive of outcomes such as readmission and discharge disposition.

## Notes

Supplement. **eAppendix 1.** Study Criteria and Patient Flow Chart **eAppendix 2.** Parameters in a Patient’s Ambulation Profile **eFigure 1.** Timeline and Ambulation History for Patient 83 **eTable 1.** Ambulation Profile for Patient 83 **eFigure 2.** Timeline and Ambulation History for Patient 92 **eTable 2.** Ambulation Profile for Patient 92 **eTable 3.** Summary of Ambulation Parameters for 100 Patients (Total Days on the PCU) **eTable 4.** Summary of Ambulation Data for 100 Patients (Full Days on the PCU) **eFigure 3.** Comparison of Ambulation Parameters for 30-Day Readmission and Discharge to Acute/Subacute Rehab **eTable 5.** Summary of *F* and *P* Values for Comparison of Outcomes Between Groups (Readmission Yes/No; Discharge Location Home/Acute Rehab) for Each of the 19 Ambulation Parameters and Length of Stay **eFigure 4.** Examples of Ambulation Distance (*d_n_*), Time (*t_n_*), and Speed (*s_n_*) Versus Ambulation Number (*n*) **eFigure 5.** Comparison of Ambulation Distance (*d_n_*), Time (*t_n_*), and Speed (*s_n_*) Versus Ambulation Number (*n*) and Time on the PCU (Beginning at 00:00 on the Transfer Day) **eTable 6.** Comparison of Prediction Models for 30-Day Readmission and Discharge Location Based on Total Days and Full Days on the PCU **eTable 7.** Prediction of Length of Stay (LoS)
