# Supplementary material for: Assessment of Patient Ambulation Profiles to Predict Hospital Readmission, Discharge Location, and Length of Stay in a Cardiac Surgery Progressive Care Unit
Source: JAMA Netw Open. 2020 Mar 17;3(3):e201074. doi: 10.1001/jamanetworkopen.2020.1074 (PMC7078761; doi:10.1001/jamanetworkopen.2020.1074)
Supplement: Supplement. — eAppendix 1. Study Criteria and Patient Flow Chart eAppendix 2. Parameters in a Patient’s Ambulation Profile eFigure 1. Timeline and Ambulation History for Patient 83 eTable 1. Ambulation Profile for Patient 83 eFigure 2. Timeline and Ambulation History for Patient 92 eTable 2. Ambulation Profile for Patient 92 eTable 3. Summary of Ambulation Parameters for 100 Patients (Total Days on the PCU) eTable 4. Summary of Ambulation Data for 100 Patients (Full Days on the PCU) eFigure 3. Comparison of Ambulation Parameters for 30-Day Readmission and Discharge to Acute/Subacute Rehab eTable 5. Summary of F and P Values for Comparison of Outcomes Between Groups (Readmission Yes/No; Discharge Location Home/Acute Rehab) for Each of the 19 Ambulation Parameters and Length of Stay eFigure 4. Examples of Ambulation Distance (dn), Time (tn), and Speed (sn) Versus Ambulation Number (n) eFigure 5. Comparison of Ambulation Distance (dn), Time (tn), and Speed (sn) Versus Ambulation Number (n) and Time on the PCU (Beginning at 00:00 on the Transfer Day) eTable 6. Comparison of Prediction Models for 30-Day Readmission and Discharge Location Based on Total Days and Full Days on the PCU eTable 7. Prediction of Length of Stay (LoS) [file jamanetwopen-3-e201074-s001.pdf]

Supplementary Online Content

Jeong IC, Healy R, Bao B, et al. Assessment of patient ambulation profiles to predict hospital readmission, discharge location, and length of stay in a cardiac surgery progressive care unit. *JAMA Netw Open.* 2020;3(3):e201074. doi:10.1001/jamanetworkopen.2020.1074

eAppendix 1. Study Criteria and Patient Flow Chart
eAppendix 2. Parameters in a Patient's Ambulation Profile
eFigure 1. Timeline and Ambulation History for Patient 83
eTable 1. Ambulation Profile for Patient 83
eFigure 2. Timeline and Ambulation History for Patient 92
eTable 2. Ambulation Profile for Patient 92
eTable 3. Summary of Ambulation Parameters for 100 Patients (Total Days on the PCU)
eTable 4. Summary of Ambulation Data for 100 Patients (Full Days on the PCU)
eFigure 3. Comparison of Ambulation Parameters for 30-day Readmission and Discharge to Acute/Subacute Rehab
eTable 5. Summary of *F* and *P* Values for Comparison of Outcomes Between Groups (Readmission Yes/No; Discharge Location Home/Acute Rehab) for Each of the 19 Ambulation Parameters and Length of Stay
eFigure 4. Examples of Ambulation Distance (d_n), Time (t_n), and Speed (s_n) Versus Ambulation Number (n)
eFigure 5. Comparison of Ambulation Distance (d_n), Time (t_n), and Speed (s_n) Versus Ambulation Number (n) and Time on the PCU (Beginning at 00:00 on the Transfer Day)
eTable 6. Comparison of Prediction Models for 30-Day Readmission and Discharge Location Based on Total Days and Full Days on the PCU
eTable 7. Prediction of Length of Stay (LoS)

This supplementary material has been provided by the authors to give readers additional information about their work.

© 2020 Jeong IC et al. *JAMA Network Open.*

eAppendix 1. Study Criteria and Patient Flow Chart.

Study criteria: patients who underwent surgery from 8/29/16 to 4/4/18
Inclusion criteria included individuals undergoing coronary artery bypass graft surgery (CABG) and/or valve surgery, aortic surgery, and/or myectomy. Exclusion criteria included lung or heart transplant, or planned insertion of ventricular assist device.

Patient Flow Chart
1066 patients screened
- 307 research staff were unavailable
- 160 surgeon was not on the IRB
- 134 declined
- 105 monitoring equipment for parent study was not available
- 38 patients were not available or schedule changed
- 36 were non-english speaking
- 17 other
- 5 could not give informed consent
- 4 ineligible surgery
- 2 target recruitment for the week was reached

238 patients consented
    patients were consented on the day of surgery or prior to surgery if inpatient
187/238 patients had IR badge detected when transferred to PCU
    51 patients did not have badge on transfer to PCU (lost badge or were excluded for other reasons)
139/238 patients had IR badge on discharge from PCU
    48 patients did not have badge at discharge (lost badge, refused to wear badge, or other reasons)
100/238 patients with at least one ambulation during stay on unit
    39 patients excluded (no ambulation data, transfer to OR/ICU, room change)

Demographic data. Data represent mean (±SD).

		30-day Readmission		Discharge to Acute Rehab	
**Ambulation Characteristics**	**All (N = 100)**	**No (N = 79)**	**Yes (N = 21)**	**No (N = 89)**	**Yes (N = 11)**
Age, mean (SD), years	63.1 (11.6)	62.1 (11.7)	66.7 (10.7)	62.3 (11.8)	68.9 (8.4)
Sex and race, Number (%)					
Women	19 (19.0)	13 (16.5)	6 (28.6)	18 (20.2)	1 (9.1)
Caucasian	14 (73.7)	11 (84.6)	3 (50.0)	14 (77.8)	
African American	4 (21.1)	1 (7.7)	3 (50.0)	3 (16.7)	1 (100)
Hispanic	1 (5.2)	1 (7.7)		1 (5.6)	
Indian (Asian)					
Other					
Men	81 (81.0)	66 (83.5)	15 (71.4)	71 (79.8)	10 (90.9)
Caucasian	68 (84.0)	55 (83.3)	13 (86.7)	61(85.9)	7 (70.0)
African American	7 (8.6)	7 (10.6)		5 (7.0)	2 (20.0)
Hispanic	1 (1.2)	1 (1.5)		1 (1.4)	
Indian (Asian)	3 (3.7)	1 (1.5)	2 (13.3)	2 (2.8)	1 (10.0)
Other	2 (2.5)	2 (3.0)		2 (2.8)	

© 2020 Jeong IC et al. *JAMA Network Open.*

eAppendix 2. Parameters in a Patient's Ambulation Profile. These parameters are determined based on the total (integer) number of days on the PCU. To account for the fact that the transfer day and discharge day usually reduced the opportunity for ambulation, parameters were calculated based on the total number of days on the PCU and the total number of days excluding the transfer and discharge days (full days). Therefore, each ambulation profile contains 19 ambulation parameters + 1 length of stay parameter = 20 parameters for either the total number of days or full days on the PCU. These parameters are defined below.

1. Total number of ambulations, *N*. The total number of ambulations completed during a patient's stay on the PCU. An ambulation is arbitrarily defined when a patient's badge is detected by at least 6 ceiling sensors in the corridor before returning to the patient's room. As for all parameters below, the total number of ambulations is calculated for total number of days or total number of days excluding transfer and discharge days.
2. Ambulation frequency (*N*/*LoS*). The total number of ambulations during a patient's stay on the PCU divided by the length of stay.
3. Number of days with ambulations (#). The number of days on the PCU where the patient completed at least one ambulation.

Compliance, %
4. % days with 1 per day. Percentage of days on the PCU where the patient completed one ambulation.
5. % days with 2 per day. Percentage of days on the PCU where the patient completed two ambulations.
6. % days with 3 per day. Percentage of days on the PCU where the patient completed three ambulations.

Ambulation distance, *d*
7. Total distance. Total distance of all ambulations completed by the patient during the patient's stay on the PCU.
8. Longest ambulation. The distance of the longest ambulation completed during the patient's stay on the PCU.
9. Mean ambulation distance (all ambulations). Mean distance of all ambulations completed during the patient's stay on the PCU.
10. Shortest ambulation. The distance of the shortest ambulation completed during the patient's stay on the PCU.

Ambulation duration, *t*
11. Longest ambulation. The duration of the longest ambulation completed during the patient's stay on the PCU.
12. Mean ambulation duration. Mean ambulation duration for all ambulations completed during the patient's stay on the PCU.
13. Shortest ambulation. The duration of the shortest ambulation completed during the patient's stay on the unit.

Ambulation speed, *s*
14. Maximum mean speed in a single ambulation. The maximum speed of a single ambulation completed during the patient's stay on the PCU. Determined from the mean speed of each ambulation.
15. Mean speed (all ambulations). The mean speed of all ambulations completed during the patient's stay on the PCU. Determined from the mean speed of each ambulation.
16. Minimum mean speed in a single ambulation. The minimum speed of a single ambulation completed during the patient's stay on the PCU. Determined from the mean speed of each ambulation.

Derivatives
17. Change in ambulation distance (d_n/n). The slope of ambulation distance versus ambulation number during the patient's stay on the PCU. $n = 1, 2, \ldots, N$. The slope was determined from a linear least squares fit.
18. Change in ambulation duration (t_n/n). The slope of ambulation duration versus ambulation number during the patient's stay on the PCU. The slope was determined from a linear least squares fit.
19. Change in ambulation mean speed (s_n/n). The slope of ambulation speed versus ambulation number during the patient's stay on the PCU. The slope was determined from a linear least squares fit.

Other
20. Length of stay, LoS. The total number of days on the PCU. The number of full days on the PCU is LoS - 2 (transfer and discharge days).

Abbreviation key
n – ambulation number ($n = 1, 2, \ldots, N$)
N – total number of ambulations
d – ambulation distance (ft)
t – ambulation time (minutes)
s – ambulation speed (m/s)

© 2020 Jeong IC et al. *JAMA Network Open.*

eFigure 1. **Timeline and Ambulation History for Patient 83.** This individual was on the PCU for 4 days and completed 2 voluntary ambulations on day 1, three ambulations on day 2, three ambulations on day 3, and 1 ambulation on day 4 prior to discharge. Note that this individual was on the PCU for two full days. The PCU floor plans show the path of each ambulation. Each ambulation is comprised of individual segments determined from the time stamp and location that a patient badge is detected by a ceiling sensor. The cumulative distance for each ambulation increases monotonically with time indicating that the patient walked continuously at an approximately constant speed without resting. The total distance for each ambulation increased from about 400 ft on day 1 to almost 800 ft on day 4. Note that the average speed per ambulation increased progressively from about 1 mph on day 1 to about 1.5 mph on day 4. See **eTable 1** for the ambulation profile for this patient.

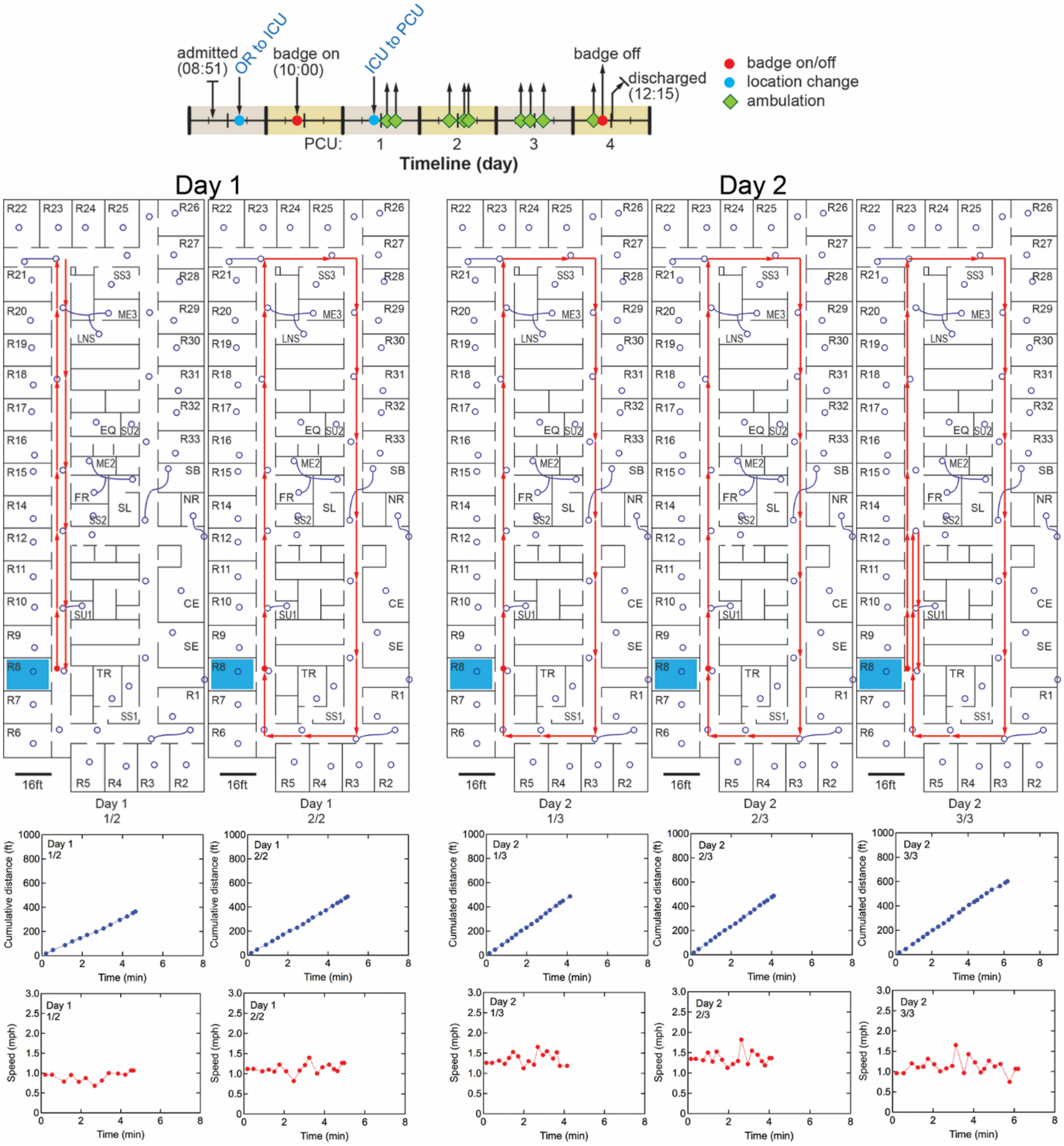

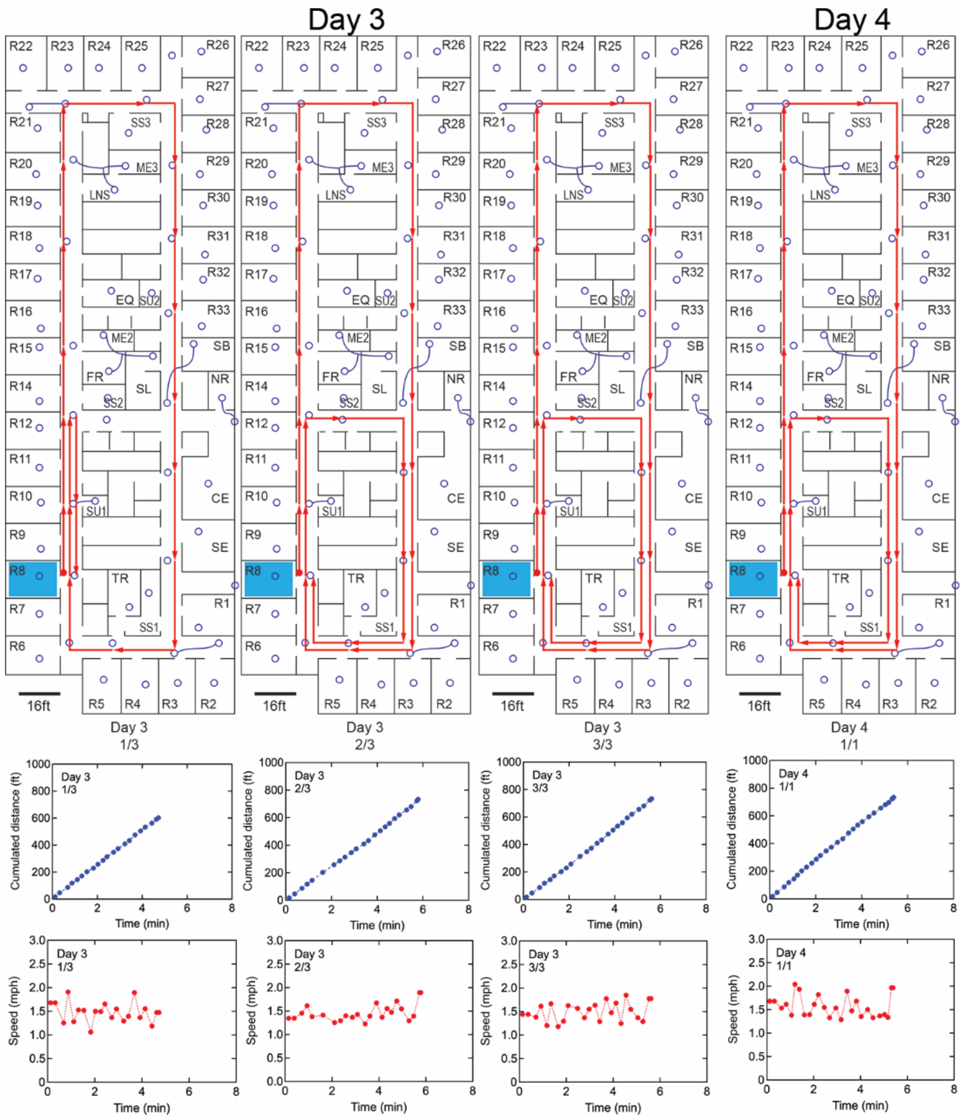

© 2020 Jeong IC et al. *JAMA Network Open.*

eTable 1. Ambulation Profile for Patient 83. Summary from all voluntary ambulations for the total number of days and full days (total days excluding transfer and discharge days) on the PCU for patient 83. This individual was discharged to home and was not readmitted. The derivatives represent a linear least squares fit to a plot of ambulation distance, ambulation duration, and mean ambulation speed versus time during an individual's stay on the PCU.

Ambulation Parameters	Total days on PCU	Full days (total minus transfer and discharge days)
LoS on PCU (days)	4	2
Total number of ambulations, *N*	9	6
Ambulation frequency (N/LoS)	2.25	3
Number of days with ambulations	4	2
Compliance, %		
% days with 1 per day	100	100
% days with 2 per day	75	100
% days with 3 per day	50	100
Ambulation distance, *d* (ft)		
Total distance	5228.8	3644.5
Longest ambulation	733.0	733.0
Mean ambulation distance (all ambulations)	581.0	607.4
Shortest ambulation	364.3	487.0
Ambulation duration, *t* (min)		
Longest ambulation	6.19	6.19
Mean ambulation duration	5.06	5.09
Shortest ambulation	4.10	4.10
Ambulation speed, *s* (m/s)		
Maximum mean speed in a single ambulation	0.69	0.67
Mean speed (all ambulations)	0.58	0.61
Minimum mean speed in a single ambulation	0.40	0.50
Derivatives		
Change in ambulation distance, d_n/n	47.0	56.2
Change in ambulation duration, t_n/n	0.146	0.312
Change in ambulation mean speed, s_n/n	0.030	0.017

© 2020 Jeong IC et al. *JAMA Network Open.*

eFigure 2. Timeline and Ambulation History for Patient 92. This individual was on the PCU for 6 days and completed four voluntary ambulations. This individual was readmitted within 30 days following discharge. See **eTable 2** for the ambulation profile for this patient.

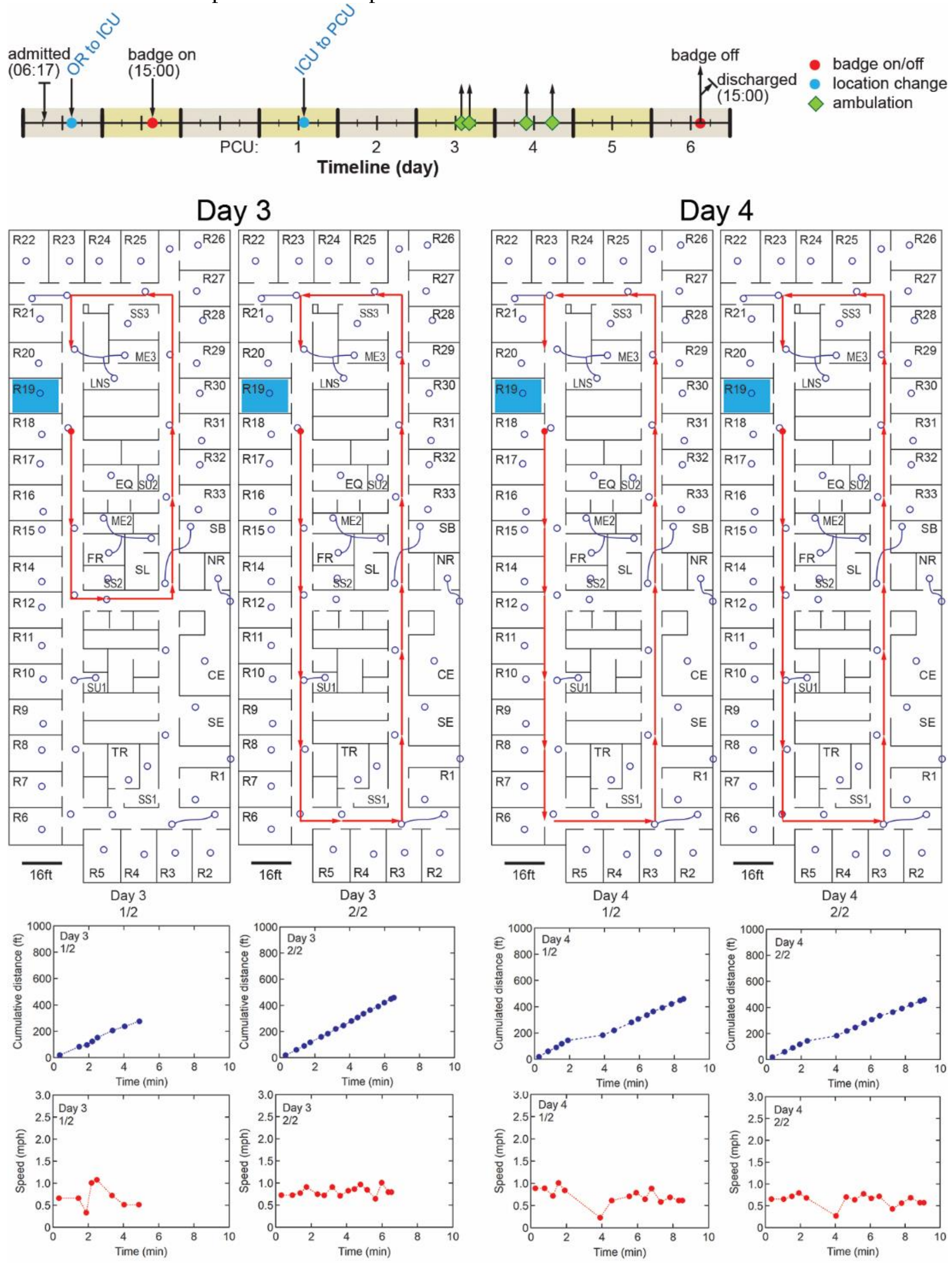

© 2020 Jeong IC et al. *JAMA Network Open.*

eTable 2. Ambulation Profile for Patient 92. Summary from all voluntary ambulations for the total number of days and full days (total days excluding transfer and discharge days) on the PCU for patient 92. This individual was on the PCU for 6 days and was readmitted 13 and 27 days after discharge. Note that the patient did not complete any ambulations on the transfer and discharge days, and hence all ambulation parameters except ambulation frequency and compliance are the same for total days and full days. The derivatives represent a linear least squares fit to a plot of ambulation distance, ambulation duration, and mean ambulation speed versus time during an individual's stay on the PCU.

**Ambulation Parameters**	**Total days on PCU**	**Full days (total minus transfer and discharge days)**
LoS on PCU (days)	6	4
Total number of ambulations, *N*	4	4
Ambulation frequency (N/LoS)	0.67	1
Number of days with ambulations	2	2
Compliance, %		
% days with 1 per day	33.3	50
% days with 2 per day	33.3	50
% days with 3 per day	0	0
Ambulation distance, *d* (ft)		
Total distance	1658.4	1658.4
Longest ambulation	460.6	460.6
Mean ambulation distance (all ambulations)	414.6	414.6
Shortest ambulation	276.5	276.5
Ambulation duration, *t* (min)		
Longest ambulation	9.06	9.06
Mean ambulation duration	7.24	7.24
Shortest ambulation	4.87	4.87
Ambulation speed, *s* (m/s)		
Maximum mean speed in a single ambulation	0.36	0.36
Mean speed (all ambulations)	0.30	0.30
Minimum mean speed in a single ambulation	0.26	0.26
Derivatives		
Change in ambulation distance, d_n/n	55.2	55.2
Change in ambulation duration, t_n/n	1.457	1.457
Change in ambulation mean speed, s_n/n	-0.017	-0.017

© 2020 Jeong IC et al. *JAMA Network Open.*

eTable 3. Summary of Ambulation Parameters for 100 Patients (Total Days on the PCU).
Analysis based on ambulation data for the total number of days on the unit. Data represent mean (± SD).

		30-day Readmission		Discharge to Acute Rehab.	
**Ambulation Characteristics**	**All (N = 100)**	**No (N = 79)**	**Yes (N = 21)**	**No (N= 89)**	**Yes (N = 11)**
LoS on PCU (days)	5.9 (2.2)	5.9 (2.3)	6.0 (1.7)	5.6 (1.9)	8.4 (3.0)
Total number of ambulations, *N*	8.4 (5.5)	8.9 (5.6)	6.3 (4.9)	8.3 (5.0)	9.2 (8.7)
Ambulation frequency (*N*/*LoS*)	1.5 (0.9)	1.6 (0.9)	1.0 (0.7)	1.5 (0.8)	1.1 (1.0)
Number of days with ambulations	3.8 (1.7)	4.0 (1.6)	2.9 (1.9)	3.7 (1.6)	4.1 (2.9)
Compliance, %					
% days with 1 per day	66.5 (23.7)	71.7 (20.9)	46.8 (23.8)	68.5 (21.7)	50.2 (33.0)
% days with 2 per day	43.4 (25.7)	46.4 (26.1)	32.1 (20.9)	45.0 (25.1)	30.7 (28.0)
% days with 3 per day	22.9 (23.4)	25.7 (24.2)	12.1 (16.8)	23.5 (23.8)	17.5 (20.4)
Ambulation distance, *d* (ft)					
Total distance	4194 (4523)	4644 (4880)	2501 (2139)	4268 (4629)	3593 (3685)
Longest ambulation	831 (1371)	905 (1520)	549 (438)	874 (1446)	482 (245)
Mean ambulation distance (all ambulations)	443 (247)	464 (266)	365 (134)	453 (255)	365 (164)
Shortest ambulation	253 (116)	258 (120)	234 (101)	255 (113)	233 (143)
Ambulation duration, *t* (min)					
Longest ambulation	11.7 (9.5)	12.4 (10.1)	9.4 (6.4)	12.0 (9.7)	9.9 (7.8)
Mean ambulation duration	6.5 (2.5)	6.7 (2.3)	5.8 (3.1)	6.5 (2.4)	6.2 (3.3)
Shortest ambulation	3.9 (1.8)	4.0 (1.7)	3.6 (1.9)	3.9 (1.5)	4.1 (3.3)
Ambulation speed, *s* (m/s)					
Maximum mean speed in a single ambulation	0.53 (0.21)	0.54 (0.21)	0.47 (0.24)	0.54 (0.22)	0.40 (0.22)
Mean speed (all ambulations)	0.38 (0.14)	0.38 (0.14)	0.37 (0.17)	0.38 (0.14)	0.31 (0.14)
Minimum mean speed in a single ambulation	0.23 (0.13)	0.22 (0.12)	0.25 (0.15)	0.23 (0.13)	0.20 (0.14)
Derivatives					
Change in ambulation distance, d_n/n	35.3 (60.6)	38.8 (61.2)	22.3 (57.8)	35.9 (62.6)	30.7 (42.9)
Change in ambulation duration, t_n/n	-0.01 (1.17)	-0.04 (1.15)	0.12 (1.25)	-0.01 (1.20)	0.04 (0.86)
Change in ambulation mean speed, s_n/n	0.023 (0.046)	0.028 (0.047)	0.003 (0.035)	0.025 (0.047)	0.007 (0.028)

© 2020 Jeong IC et al. *JAMA Network Open.*

eTable 4. Summary of Ambulation Data for 100 Patients (Full Days on the PCU). Analysis based on ambulation data for the number of full days on the unit (total days excluding transfer and discharge days). Data represent mean (± SD).

		30-day Readmission		Discharge to Acute Rehab.	
**Ambulation Characteristics**	**All (n = 100)**	**No (n = 79)**	**Yes (n = 21)**	**No (n = 89)**	**Yes (n = 11)**
LoS on PCU (days)					
Total number of ambulations, *N*	7.0 (4.9)	7.4 (5.1)	5.3 (3.9)	6.9 (4.4)	8.1 (8.1)
Ambulation frequency, *N*/*LoS*	2.0 (1.1)	2.1 (1.1)	1.3 (0.8)	2.0 (1.1)	1.4 (1.2)
Number of days with ambulations	3.0 (1.6)	3.1 (1.6)	2.4 (1.6)	2.9 (1.5)	3.5 (2.5)
Compliance, %					
% days with 1 per day	82.9 (25.1)	88.4 (20.8)	61.9 (28.9)	85.9 (21.4)	58.4 (37.9)
% days with 2 per day	61.1 (35.3)	65.7 (35.1)	44.0 (31.0)	63.8 (34.2)	39.4 (38.0)
% days with 3 per day	33.7 (36.5)	38.5 (38.3)	15.8 (20.8)	35.0 (37.5)	23.0 (25.0)
Ambulation distance, *d* (ft)					
Total distance	3458 (3882)	3832 (4221)	2050 (1566)	3493 (3941)	3176 (35115)
Longest ambulation	779 (1357)	857 (1514)	487 (230)	816 (1432)	479 (248)
Mean ambulation distance (all ambulations)	449 (280)	471 (304)	368 (136)	460 (290)	362 (163)
Shortest ambulation	269 (127)	276 (133)	244 (102)	273 (126)	233 (143)
Ambulation duration, *t* (min)					
Longest ambulation	11.1 (9.3)	12.0 (10.1)	7.9 (4.4)	11.3 (9.5)	9.8 (7.8)
Mean ambulation duration	6.6 (2.6)	6.8 (2.5)	5.6 (3.0)	6.6 (2.5)	6.2 (3.4)
Shortest ambulation	4.1 (1.8)	4.2 (1.8)	3.7 (1.9)	4.1 (1.6)	4.1 (3.3)
Ambulation speed, *s* (m/s)					
Maximum mean speed in a single ambulation	0.51 (0.21)	0.52 (0.20)	0.46 (0.24)	0.52 (0.21)	0.46 (0.21)
Mean speed (all ambulations)	0.38 (0.15)	0.38 (0.14)	0.37 (0.18)	0.38 (0.15)	0.37 (0.14)
Minimum mean speed in a single ambulation	0.25 (0.14)	0.24 (0.14)	0.28 (0.15)	0.24 (0.14)	0.28 (0.14)
Derivatives					
Change in ambulation distance, d_n/n	43.0 (88.6)	46.1 (89.9)	31.7 (84.5)	43.5 (92.2)	39.3 (52.5)
Change in ambulation duration, t_n/n	0.01 (1.36)	-0.05 (1.41)	0.26 (1.19)	0.00 (1.41)	0.09 (0.93)
Change in ambulation mean speed, s_n/n	0.027 (0.063)	0.031 (0.061)	0.010 (0.058)	0.031 (0.063)	0.010 (0.030)

© 2020 Jeong IC et al. *JAMA Network Open.*

eFigure 3. Comparison of Ambulation Parameters for 30-Day Readmission and Discharge to Acute/Subacute Rehab. Bars represent mean ± 95% confidence limits. Comparison based on total days on the unit. 0 – not readmitted or not discharged to acute/subacute rehab. 1 - readmitted or discharged to acute/subacute rehab. N = 100.

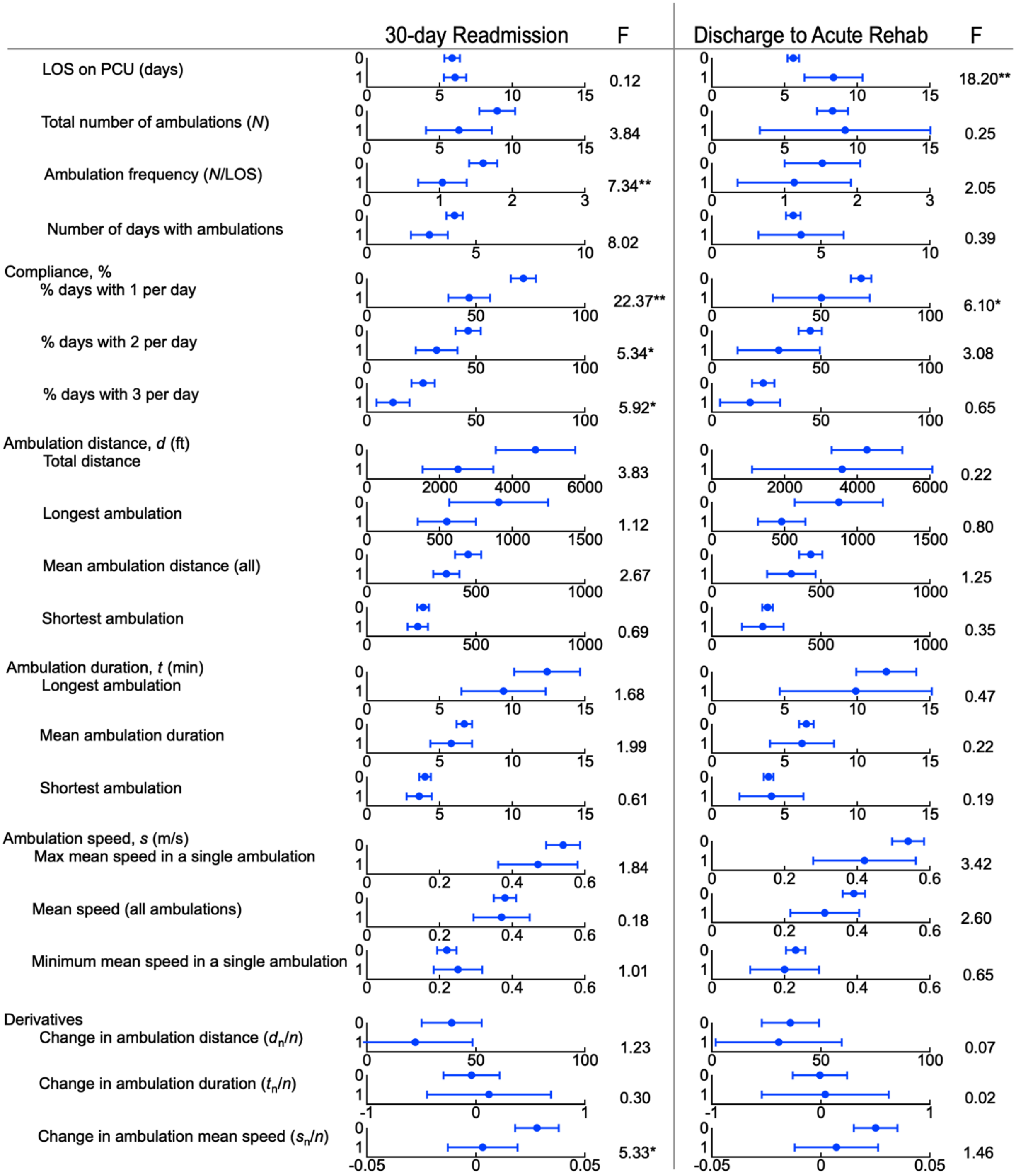

© 2020 Jeong IC et al. *JAMA Network Open.*

eTable 5. Summary of *F* and *P* Values for Comparison of Outcomes Between Groups (Readmission Yes/No; Discharge Location Home/Acute Rehab) for Each of the 19 Ambulation Parameters and Length of Stay. Ambulation data for 100 patients (total days on the PCU). Threshold values were determined in the following way. If there was no overlap of the ±95% confidence limits between the two groups then the threshold was determined from the average of the lower and higher values of each group. If the bands overlapped then the threshold was determined as the average of the mean values of the two groups.

	30-day Readmission				Discharge to Acute Rehab.			
**Ambulation Characteristics**	***threshold***	***F***	***p***	***F&p***	***threshold***	***F***	***p***	***F&p***
LoS on PCU (days)	6.0	0.12	0.731		7.8	18.2	4.6×10^{-6}	
Total number of ambulations, *N*	8.1	3.84	0.053		11.1	0.25	0.62	
Ambulation frequency (*N*/*LoS*)	1.4	7.34	0.008		1.6	2.05	0.16	
Number of days with ambulations	3.7	8.02	0.006		4.7	0.39	0.53	
Compliance, %								
% days with 1 per day	62.3	22.4	7.5×10^{-6}		68.2	6.1	0.015	
% days with 2 per day	41.1	5.3	0.023		44.6	3.1	0.082	
% days with 3 per day	20.0	5.9	0.017		24.8	0.65	0.42	
Ambulation distance, *d* (ft)								
Total distance	3513	3.8	0.053		4681	0.65	0.64	
Longest ambulation	657	1.1	0.29		608	0.80	0.37	
Mean ambulation distance (all ambulations)	415	2.7	0.11		437	1.3	0.27	
Shortest ambulation	256	0.69	0.41		280	0.35	0.56	
Ambulation duration, *t* (min)								
Longest ambulation	11.2	1.7	0.20		12.5	0.47	0.50	
Mean ambulation duration	6.7	2.0	0.16		7.2	0.22	0.64	
Shortest ambulation	4.0	0.62	0.44		4.9	0.19	0.66	
Ambulation speed, *s* (m/s)								
Maximum mean speed in a single ambulation	1.20	1.8	0.18		1.18	3.4	0.067	
Mean speed (all ambulations)	0.89	0.18	0.67		0.85	2.6	0.11	
Minimum mean speed in a single ambulation	0.57	1.0	0.32		0.56	0.65	0.42	
Derivatives								
Change in ambulation distance, d_n/n	36.8	1.2	0.27		41.1	0.07	0.79	
Change in ambulation duration, t_n/n	0.19	0.30	0.58		0.17	0.02	0.89	
Change in ambulation mean speed, s_n/n	0.04	5.3	0.023		0.05	1.46	0.23	

F and *p* values for ambulation parameters associated with 30-day readmission (yes/no) and discharge location (yes/no):

$p<0.01$ $p<0.05$ $p\geq0.05$&$F>3$ $2<F\leq3$ $1<F\leq2$ $F\leq1$

© 2020 Jeong IC et al. *JAMA Network Open.*

eFigure 4. Examples of Ambulation Distance (d_n), Time (t_n), and Speed (s_n) Versus Ambulation Number (n). The derivatives d_n/n, t_n/n, and s_n/n, provide information on the changes in a patient's mobility during recovery on the PCU. The slopes of these plots, determined from a linear least squares fit ,were included in each patient's ambulation profile. (A) Profiles for patients P7, P74, and P83 (not readmitted within 30-days), (B) profiles for patients P37, P70, and P92 (readmitted within 30 days of discharge). P7 completed twelve voluntary ambulations but showed no obvious trend in terms of distance. However, the duration of each ambulation decreased corresponding to a monotonic increase in ambulation speed. P74 completed six ambulations but with only a small increase in distance over time. However, the decrease in duration also resulted in a monotonic increase in average speed. P83 completed nine ambulations with a monotonic increase in distance in approximately the same duration, corresponding to an increase in average speed. The three individuals who were readmitted within 30 days showed small increases in distance and duration, but the corresponding average speed was either constant or decreased with number of ambulations.

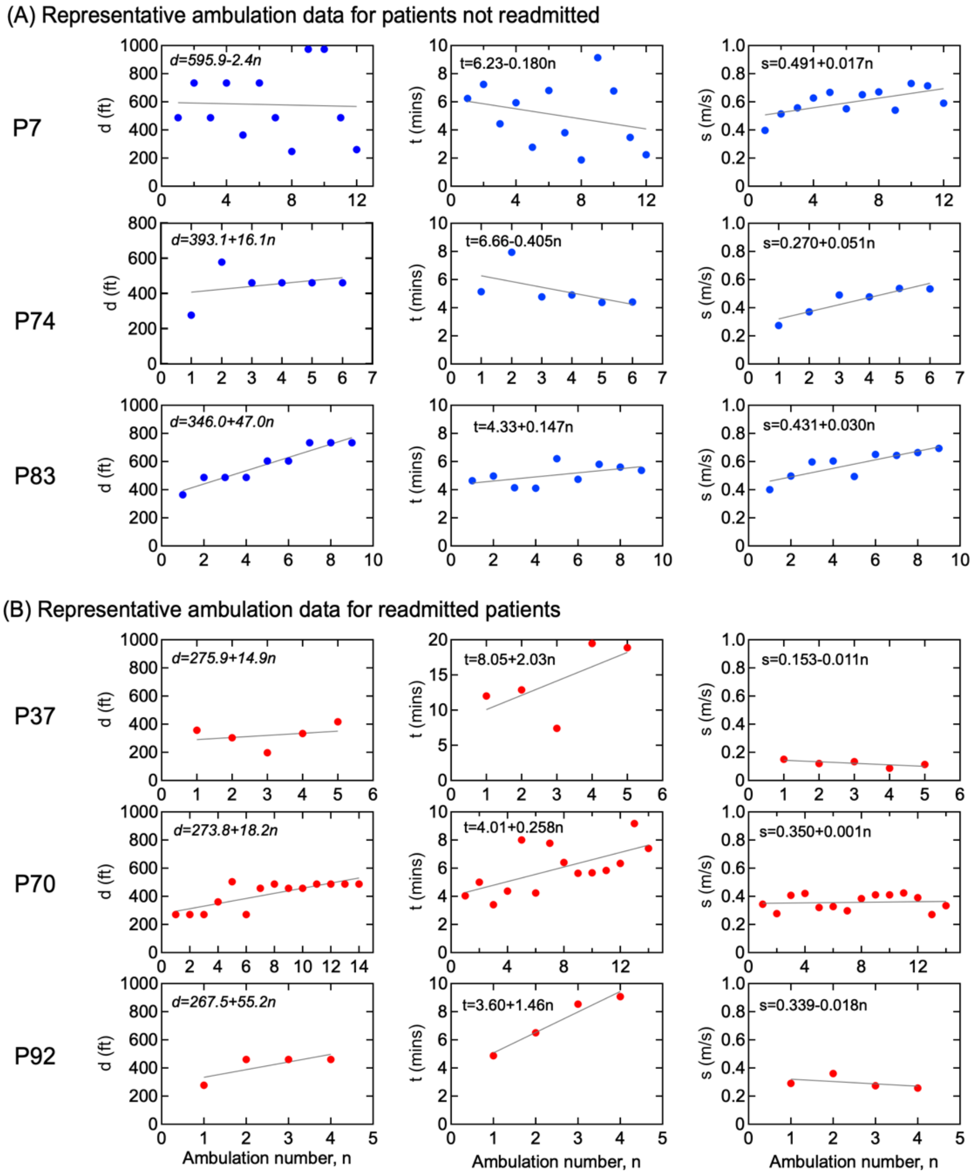

© 2020 Jeong IC et al. *JAMA Network Open.*

eFigure 5. Comparison of Ambulation Distance (d_n), Time (t_n), and Speed (s_n) Versus Ambulation Number (n) and Time on the PCU (Beginning at 00:00 on the Transfer Day). The changes in d_n, t_n, and s_n can be defined in terms of the number of ambulations (d_n/n, t_n/n, and s_n/n) or in terms of the time on the unit (d_n/t, t_n/t, and s_n/t) beginning at 00:00 on the transfer day. To assess whether there was a difference, we determined the derivatives for both the ambulation number and time on the PCU. (A) Distance, duration, and average speed versus ambulation number and time on the PCU for patient 83 who was discharged to home and was not readmitted within 30 days. (B) Comparison of change in ambulation speed determined from ambulation number (s_n/n) and time on the PCU (s_n/t) for all patients (N = 100) based on whether they were readmitted within 30 days. Bars represent mean ± 95% confidence limits. The correlation between non-readmitted and readmitted patients with change in speed per ambulation (s_n/n) is significantly larger than for change in speed per time on the PCU (s_n/t). Therefore, change in speed per ambulation (s_n/n) was used in the ambulation profiles and predictive models.

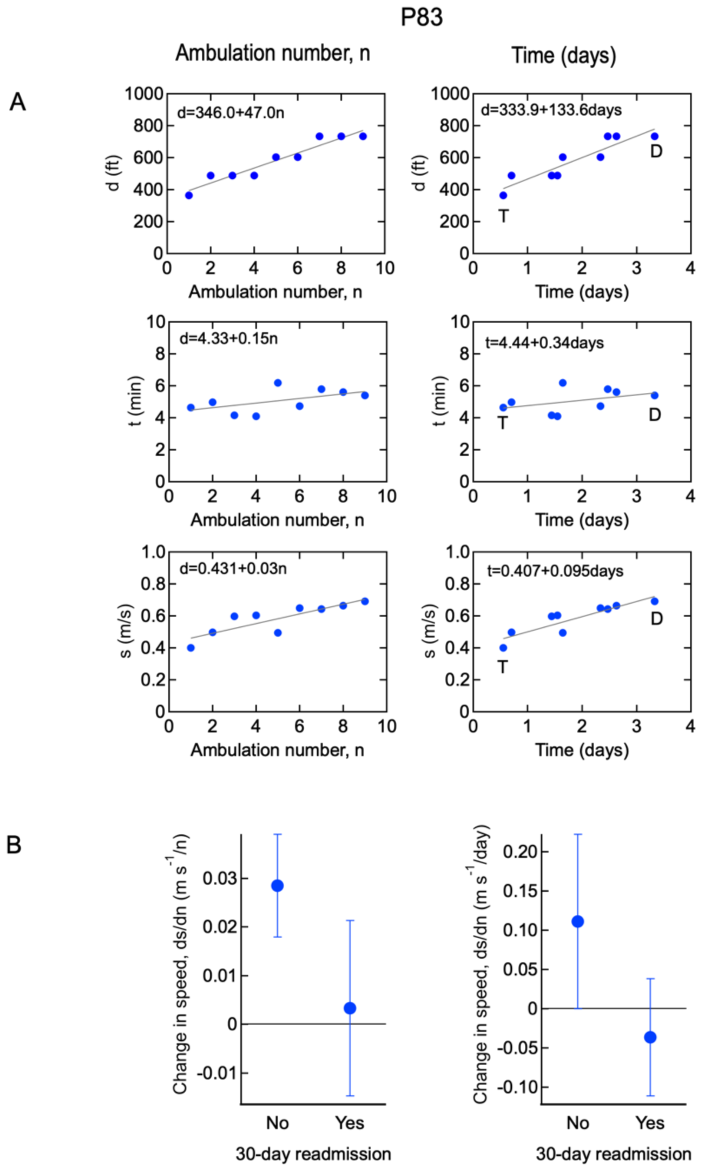

© 2020 Jeong IC et al. *JAMA Network Open.*

eTable 6. Comparison of Prediction Models for 30-Day Readmission and Discharge Location Based on Total Days and Full Days on the PCU. Models based on 19 parameters in ambulation profile. Having established that several ambulation parameters were statistically significant between 30-day readmission and discharge location groups, a stepwise binary regression model was used to assess predictions using a split sampling method (70/30). In the model based on total days, 30-day readmissions were predicted with 86.7% sensitivity and 88.2% selectivity and the discharge location was predicted with 84.6% sensitivity and 86.4% selectivity. The model predictions for 30-day readmissions improved slightly (93.3% sensitivity and 88.2% selectivity) based on full days on the PCU, but decreased for discharge location (84.6% sensitivity and 77.3% selectivity). Full days represents the number of days on the PCU excluding transfer and discharge days. Since the number of 30-day readmissions and cases of discharge to acute rehab were relatively small, we used a synthetic minority over-sampling technique (imblearn.over_sampling.SMOTE, Python) to balance the data prior to analysis.

Model based on total days on the PCU

Dependent Variables	Classification Test Result, cases (%)	
	Sensitivity	Specificity
30-day readmission (yes)	86.7%	88.2%
Discharge to acute rehab (yes)	84.6%	86.4%

PREDICTORS

30-day readmissions	β	SE β	Wald's χ^2	df	p	e^β (odds ratio)	95% CI for e^β Lower	Upper
% days with 1 ambulation per day	-0.374	0.087	18.297	1	0.000	0.688	0.580	0.817
number of days with ambulations (*X 10*)	0.456	0.124	13.532	1	0.000	1.578	1.238	2.012
LoS on PCU	-2.728	0.734	13.809	1	0.000	0.065	0.016	0.276
total number of ambulations	0.531	0.197	7.235	1	0.007	1.700	1.155	2.503
total distance (*X 0.01*)	-0.123	0.043	8.075	1	0.004	0.884	0.812	0.962
mean ambulation duration (*X 0.01*)	0.446	0.223	4.010	1	0.045	1.563	1.010	2.419
maximum mean speed in a single ambulation	-0.363	0.150	5.879	1	0.015	0.696	0.519	0.933
mean speed (all ambulations)	0.832	0.232	12.850	1	0.000	2.298	1.458	3.623
change in ambulation mean speed (s_n/n)	-0.889	0.290	9.430	1	0.002	0.411	0.233	0.725
Constant	16.496	4.508	13.393	1	0.000			

β: coefficient, SE: coefficient standard error, χ^2: Chi-Square. CI: confidential interval

Note. Predictions based on the binary stepwise regression method, including or removing an independent variable at each step based on the p-value of *F* (Entry: 0.05, Removal: 0.10).

Final Step	Group	Observed		Predicted: 30-day readmission Yes	No	Percentage Correct
12	Training	30-day readmission	Yes	57	7	89.1
			No	12	50	80.6
		Overall Percentage				84.9
	Testing	30-day readmission	Yes	13	2	86.7
			No	2	15	88.2
		Overall Percentage				87.5

original data (Yes: 21, No: 79, total: 100), balanced data (Yes: 79, No: 79, total: 158)

© 2020 Jeong IC et al. *JAMA Network Open.*

PREDICTORS

Discharge to acute rehab	β	SE β	Wald's χ^2	df	p	e^β (odds ratio)	95% CI for e^β Lower	Upper
% days with 1 ambulation per day	-0.221	0.059	14.116	1	0.000	0.801	0.714	0.900
% days with 2 ambulations per day	-0.180	0.062	8.479	1	0.004	0.835	0.740	0.943
number of days with ambulations *(X 10)*	0.373	0.091	16.879	1	0.000	1.452	1.215	1.735
ambulation frequency *(X 10)*	1.294	0.376	11.864	1	0.001	3.647	1.746	7.615
total distance	-0.002	0.001	10.720	1	0.001	0.998	0.996	0.999
longest ambulation distance	-0.015	0.004	11.509	1	0.001	0.985	0.977	0.994
mean ambulation distance (all ambulations)	0.073	0.018	16.109	1	0.000	1.076	1.038	1.115
mean ambulation duration	-0.039	0.011	13.345	1	0.000	0.962	0.942	0.982
mean speed (all ambulations) *(X100)*	-0.481	0.129	13.986	1	0.000	0.618	0.480	0.795
change in ambulation distance (d_n/n)	0.089	0.026	11.828	1	0.001	1.093	1.039	1.150
change in ambulation duration (t_n/n)	-0.044	0.017	6.739	1	0.009	0.957	0.925	0.989
change in ambulation mean speed (s_n/n) *(X100)*	-0.909	0.275	10.958	1	0.001	0.403	0.235	0.690
Constant	5.902	2.816	4.393	1	0.036			

Note. Predictions based on the binary stepwise regression method, including or removing an independent variable at each step based on the p-value of *F* (Entry: 0.05, Removal: 0.10).

Final Step	Group	Observed		Predicted: Discharge to acute rehab Yes	No	Percentage Correct
9	Training	Discharge to acute rehab	Yes	57	6	90.5
			No	8	59	88.1
		Overall Percentage				89.2
	Testing	Discharge to acute rehab	Yes	22	4	84.6
			No	3	19	86.4
		Overall Percentage				85.4

original data (Yes: 11, No: 89, total: 100), balanced data (Yes: 89, No: 89, total: 178)

© 2020 Jeong IC et al. *JAMA Network Open.*

Model based on full days on the PCU

Dependent Variables	Classification Test Result, cases (%)	
	Sensitivity	Specificity
30-day readmission	93.3%	88.2%
Discharge to acute rehab	84.6%	77.3%

PREDICTORS

30-day readmissions: LoS on PCU, ambulation frequency, number of days with ambulations, % days with 1 ambulation per day, % days with 3 ambulations per day, total distance, mean ambulation distance (all ambulations), shortest ambulation distance, maximum mean speed in a single ambulation, mean speed (all ambulations), change in ambulation duration (t_n/n).

Discharge to acute rehab: ambulation frequency, number of days with ambulations, % days with 1 ambulation per day, % days with 2 ambulations per day, total distance, longest ambulation distance, mean ambulation distance (all ambulations), shortest ambulation duration, mean ambulation duration, mean speed (all ambulations), change in ambulation distance (d_n/n)

Note. Predictions based on the binary stepwise regression method, including or removing an independent variable at each step based on the p-value of F (Entry: 0.05, Removal: 0.10).

Final Step	Group	Observed		Predicted: 30-day readmission Yes	Predicted: 30-day readmission No	Percentage Correct
10	Training	30-day readmission	Yes	60	4	93.8
			No	8	54	87.1
		Overall Percentage				90.5
	Testing	30-day readmission	Yes	14	1	93.3
			No	2	15	88.2
		Overall Percentage				90.6

original data (Yes: 21, No: 79, total: 100), balanced data (Yes: 79, No: 79, total: 158)

Final Step	Group	Observed		Predicted: Discharge to acute rehab Yes	Predicted: Discharge to acute rehab No	Percentage Correct
10	Training	Discharge to acute rehab	Yes	56	7	88.9
			No	11	56	83.6
		Overall Percentage				86.2
	Testing	Discharge to acute rehab	Yes	22	4	84.6
			No	5	17	77.3
		Overall Percentage				81.3

original data (Yes: 11, No: 89, total: 100), balanced data (Yes: 89, No: 89, total: 178)

© 2020 Jeong IC et al. *JAMA Network Open.*

eTable 7. Prediction of Length of Stay (LoS). Prediction models were generated using stepwise binary regression with split sampling (70/30). Models based on 19 parameters in the ambulation profile. Full days represents the number of days on the PCU excluding transfer and discharge days. The correlation coefficient for predicted length of stay was 0.927 for total days and 0.922 for full days on the unit.

Total days on PCU

Dependent Variable	Group	Coefficient of Correlation	Coefficient of Determination
LoS on PCU	Training	0.943	0.888
	Testing	0.927	

Predictors	β*	SE	Std. β	t	p
Constant	6.534	0.313		20.908	0.000[a]
Number of days with ambulations	1.313	0.064	1.070	20.550	0.000[b]
% days with 1 ambulation per day, shortest ambulation	-0.083	0.005	-0.873	-16.776	0.000[c]
change in ambulation mean speed (s_n/n).	-2.214	0.762	-0.126	-2.904	0.005

β*: unstandardized coefficient, SE: coefficient standard error, Std.β: standardized coefficients, a = 3.097E-29, b = 7.647E-29, c =2.493E-24

Note. Correlation based on linear stepwise regression, based on the p-value of F (Entry: 0.05, Removal: 0.10).

Full days on PCU

Dependent Variable	Group	Coefficient of Correlation	Coefficient of Determination
LoS on PCU	Training	0.957	0.915
	Testing	0.922	

PREDICTORS

Number of days with ambulations, % days with 1 ambulation per day.

Note. Correlation based on linear stepwise regression, including or removing one independent variable at each step, based on the p-value of F (Entry: 0.05, Removal: 0.10).

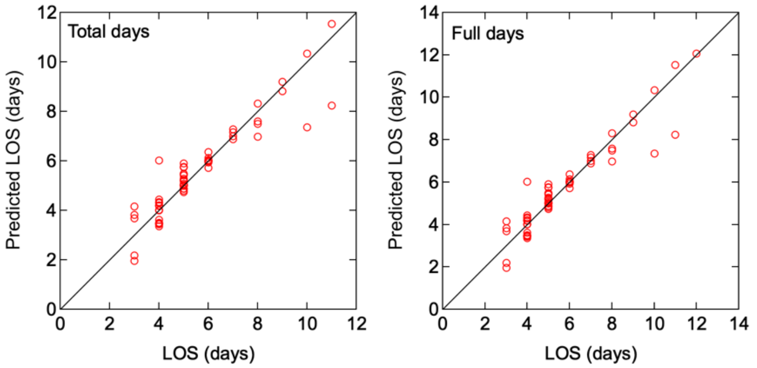

Predicted LOS for total days and full days on the unit for the test sample.

© 2020 Jeong IC et al. *JAMA Network Open.*
